# Energy localization and spatiotemporal pattern evolution mechanism of spatial thin-film structures under parametric excitation

**DOI:** 10.1371/journal.pone.0353936

**Published:** 2026-07-17

**Authors:** Teng Li, Xingjun Tong, Xinghong Liu, Dapeng Zhou

**Affiliations:** 1 School of Mechanical and Electrical Engineering, Qiqihar University, Qiqihar, Heilongjiang, China; 2 Qiqihaer Heping Heavy Industries Group Co., Ltd, Qiqihar, Heilongjiang, China; 3 School of Mechanical Engineering, Liaoning Vocational College, Tieling, Liaoning, China; 4 Harbin Boiler Factory Co., Ltd, Harbin, Heilongjiang, China; Federal University of Technology - Parana, BRAZIL

## Abstract

This paper investigates energy localization and spatiotemporal pattern evolution in spatial thin-film structures subjected to parametric excitation. A nonlinear thin-shell model is established by combining geometric nonlinearity, time-varying in-plane tension, damping, and multimodal coupling. A second-order multiple-scales perturbation procedure is then used to derive slow-flow modulation equations, and a high-fidelity finite element platform verifies the resulting localization and pattern-selection predictions. The analysis shows that pronounced localization occurs near the combined-resonance condition $\omega_{\text{1}}\;+\;\omega_{\text{2}})/\text{2}$, when the normalized tension fluctuation exceeds approximately γ > 0.20. The nonlinear modal coupling strength κ controls topology selection: stationary breathers dominate for κ < 0.35, whereas traveling patterns emerge for κ > 0.40, with a transition centered near $\kappa_{c}\;\approx \;\text{0}.\text{375}$. The pattern wavelength follows λ/L ≈ 0.28 for square and near-square membranes, while highly anisotropic geometries require direction-dependent correction. Long-term simulations over 2000 excitation cycles show decay rates below 3%, indicating attractor-like persistence within the verified numerical horizon. Frequency-wavenumber spectra further confirm selective modal amplification at resonance. The results provide quantitative criteria for pretension management, hot-spot prediction, and placement of stiffeners, dampers, or active actuators in deployable aerospace membrane structures.

## 1. Introduction

Space thin-film structures are subjected to parametric excitations generated by thermal cycling, solar-pressure perturbations, reaction-wheel disturbances, and attitude maneuvers during on-orbit operation. Such excitations induce nonlinear dynamic phenomena through periodic modulation of system stiffness [1,2]. Energy can be selectively pumped into specific modal pairs, producing vibration hot spots and spatiotemporal patterns on the structural surface [3]. This phenomenon has immediate implications for structural stiffness stability, fatigue life, functional integrity, and mission reliability of spacecraft [4,5]. Therefore, a clear understanding of the energy transport path and pattern evolution mechanism under parametric excitation is essential for high-precision dynamic design and on-orbit health management of flexible structures.

Existing theories for the dynamics of thin-film space structures do not yet fully describe the entire process of nonlinear energy redistribution in a parametrically excited system [6,7]. Most analyses focus on linear or weakly nonlinear responses to external forcing and therefore omit the special modal interaction paths and energy-transfer mechanisms generated by parametric excitation [8,9]. Periodic modulation of the stiffness or mass matrix can open originally uncoupled modal channels, leading to cross-modal energy jumps and phase locking under particular detuning conditions [10,11]. This process is not a homogeneous diffusion phenomenon; rather, it forms high-concentration energy regions in physical space, namely energy localization [12,13]. The system variables evolve synchronously in the spatial and temporal dimensions, and their morphology depends sensitively on the initial disturbance, damping distribution, and boundary conditions [14,15]. Without a rigorous separation between fast oscillatory motion and slow amplitude evolution, it is difficult to describe the competition among parametric gain, nonlinear saturation, and dissipation in the amplitude modulation equations [16,17]. In addition, stability results for differential equations with periodic coefficients often rely on numerical thresholds, while quantitative bifurcation detection based on Floquet multiplier spectra remains insufficiently connected to localization criteria [18,19]. The lack of an explicit analytical relationship between spatiotemporal pattern evolution and modal stability also limits active control and suppression strategies [20,21].

Previous studies on nonlinear vibration of thin-film structures commonly used linear modal superposition, which neglects geometric-nonlinearity-induced modal coupling and reduces the structure to a discrete degree-of-freedom model [22,23]. Galerkin truncation and harmonic-balance methods were later introduced to handle weak nonlinear terms; however, these methods are mainly suitable for small-amplitude vibration and may lose accuracy near internal resonance regions [24,25]. To describe large-deformation effects, continuous models based on von Kármán plate theory, shell theory, and direct numerical integration have been employed to obtain transient responses [26,27]. Although these methods can reproduce local high-amplitude events, they do not fully reveal the underlying energy-transfer mechanism. Invariant-manifold and center-manifold reductions provide another way to reduce system dimension [28,29], but constructing manifolds under parametric excitation is difficult because the governing operator itself is time periodic. Energy-based approaches have also been used to analyze intermodal transfer efficiency [30,31], yet they often ignore how spatially nonuniform damping redirects dissipation paths. The fundamental limitation of these classical approaches is the absence of a unified theory capable of treating multiscale dynamics, periodic-coefficient stability, and nonlinear modal coupling simultaneously.

In recent years, the combined use of multiple-scales perturbation and Floquet theory in periodic systems has received increasing attention. Some studies have separated fast and slow time scales in string vibration analysis [32,33], obtaining amplitude evolution equations and predicting modulational-instability regions of wavetrain solutions. Other studies have used Floquet multiplier spectra to identify parametric-resonance boundaries through numerical continuation of periodic solution branches [34,35]. For flexible structures, perturbation expansions combined with modal orthogonality have been used to derive nonlinear coupling coefficients [36,37], but the compatibility between secular-term removal and energy conservation constraints has not been systematically discussed. Additional work has applied Floquet theory to time-varying stiffness beams [38,39], although the influence of geometric nonlinearity on modal-shape adjustment and higher-mode participation is often underestimated. These developments show that multiple-scales analysis can separate excitation frequency from response envelopes, whereas Floquet analysis can provide rigorous linearized stability criteria; however, an explicit link between multiplier spectra, damping, nonlinear coupling, and energy-localization thresholds remains incomplete.

Beyond structural mechanics, related nonlinear resonant-interaction and energy-localization mechanisms have been reported in perturbative plasma-wave and nonlinear-flow systems, including resonant harmonic coupling, chaotic excitation of rogue waves, nonlinear surface-wave excitation, and field-theoretic momentum-transport structures [40–43]. Recent studies on parametrically excited rotating beams, vortex-induced multistability, time-varying synchronization, Chebyshev-Ritz parametric resonance, and adaptive fuzzy vibration control further demonstrate that time-varying coefficients, attractor coexistence, and control laws can substantially reshape instability tongues and vibration-energy pathways [44–48]. These related works broaden the context of the present study and motivate a framework that links resonant energy injection, nonlinear modal coupling, pattern selection, and control-oriented suppression in spatial thin-film structures.

This paper investigates the intrinsic mechanism of energy localization and spatiotemporal pattern formation in spatial thin-film structures subjected to parametric excitation. The central objective is to derive a dynamic stability criterion for the combined influence of time-varying tension and geometric nonlinearity, and then to identify the parameter region in which energy concentration occurs. A nonlinear thin-shell model containing time-dependent tension terms is combined with a second-order multiple-scales perturbation procedure to obtain complex-amplitude modulation equations. The effect of nonlinear modal coupling coefficients on the direction and rate of energy transport is then analyzed. High-accuracy finite element simulations reproduce the complete evolution route from initial perturbation to representative structures such as stationary breathers and traveling patterns, and key quantities including dominant wavenumber, propagation velocity, and localization width are extracted. In addition, an energy flux density field is formulated to examine intermodal energy exchange and to explain how parametric excitation breaks system symmetry and triggers spontaneous mode selection.

The main highlights and contributions of this work are summarized as follows:

(1) A unified thin-shell formulation is established for spatial thin-film structures under time-varying tension. The model retains geometric nonlinearity, modal coupling, weak damping, and parametric stiffness modulation, thereby connecting the continuous governing equations with the finite element discretization and the slow-flow analytical model.
(2) The physical thresholds are interpreted quantitatively: γ > 0.20 indicates the point at which cycle-averaged parametric input exceeds damping and nonlinear redistribution, whereas $\kappa_{c}\;\approx \;\text{0}.\text{375}$ separates phase-locked stationary breathers from symmetry-broken traveling patterns. The scaling relationship λ/L ≈ 0.28 is further shown to hold for square and near-square membranes.
(3) A high-fidelity transient simulation platform is used to validate the analytical predictions through RMS localization maps, phase-space reconstruction, long-term stability checks, and frequency-wavenumber spectra. The numerical evidence confirms the three-stage route from initial perturbation to hot-spot nucleation and finally to saturated localized patterns.
(4) A control-oriented implementation route is proposed by linking modal-energy maps and energy-flux fields to practical decisions on pretension retuning, local stiffener placement, passive damping, and active actuator layout. The discussion also clarifies the validity limits associated with frequency drift, flexible support frames, material memory, and highly anisotropic membranes.

## 2. Parametric excitation thin shell dynamics modeling and solution framework

**Fig 1:**
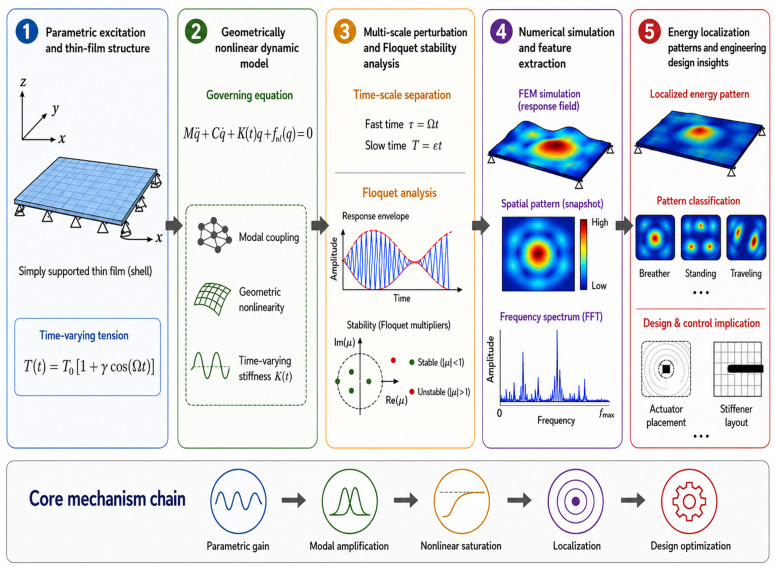
Framework for dynamic analysis of parametrically excited thin film structures.

Fig 1 shows the framework for the dynamic analysis of parametrically excited thin-film structures. At the theoretical-modeling level, nonlinear thin-shell equations with a time-dependent tension field are established. At the analytical level, amplitude modulation equations are derived by multiple-scales perturbation. The numerical-simulation level generates the full-field transient response, which is then processed by spatiotemporal Fourier transform, modal projection, and phase-space reconstruction in the feature-extraction layer. Finally, the physical-mechanism layer detects energy localization, classifies spatiotemporal patterns, and verifies stability, thereby revealing the causal chain from parametric-excitation input to complex dynamic response.

### 2.1 Constructing geometrically nonlinear control equations for thin shells with time-varying tension

To quantitatively characterize the energy-localization and pattern-evolution mechanisms under parametric excitation, it is necessary to establish dynamic governing equations that accurately describe the coupling between geometric nonlinearity and time-varying stiffness. Based on Kirchhoff-Love thin-shell theory [49,50], the displacement field is expressed through in-plane displacement components and transverse deflection while retaining the quadratic nonlinear terms of the displacement gradient. This formulation provides a geometrically nonlinear framework. The in-plane strain is written in Green-Lagrange form [51,52] as:


$$\begin{array}{c} \epsilon_{ij}=\frac{\text{1}}{\text{2}}\left(\frac{\partial u_{i}}{\partial x_{j}}+\frac{\partial u_{j}}{\partial x_{i}}+\frac{\partial w}{\partial x_{i}}\frac{\partial w}{\partial x_{j}}\right) \end{array}$$ (1)

where $\epsilon_{ij}$ is the in-plane strain component, u and v are the in-plane displacement components, w is the transverse deflection, and x and y are the spatial coordinates. The stress-strain relationship follows a linear elastic constitutive model but is solved within the nonlinear strain framework, thereby retaining the coupling between transverse bending and in-plane tension. The governing equations are obtained using the energy variational method [53,54]. The Lagrangian energy consists of the kinetic energy and the strain energy of the shell. The kinetic energy is expressed as:


$$\begin{array}{c} T=\frac{\text{1}}{\text{2}}\int_{V}^{\;}\rho \left({\dot{u}}_{i}{\dot{u}}_{i}+{\dot{w}}^{\text{2}}\right)dV \end{array}$$ (2)

where ρ is the density of the thin shell, $u_{i}$ is the in-plane velocity component, $w^{\cdot }$ is the transverse-deflection velocity, and V is the shell volume. The potential energy is given by:


$$\begin{array}{c} U=\frac{\text{1}}{\text{2}}\int_{S}^{\;}\sigma_{ij}\epsilon_{ij}dS \end{array}$$ (3)

where $\sigma_{ij}$ is the stress component, $\epsilon_{ij}$ is the strain component, and dA is the surface-element area of the thin shell. In this form, the in-plane strain energy and out-of-plane bending energy are included consistently. By applying Hamilton’s principle [55,56] to the kinetic and potential energy, the nonlinear governing equations are obtained, enabling a coupled description of transverse bending, in-plane tension, and shear stiffness.

To ensure the reproducibility of subsequent analytical developments and establish a rigorous foundation for the multi-scale perturbation analysis, the complete set of continuous governing equations is explicitly presented. The nonlinear strain-displacement relations in full component form are:


$$\begin{array}{c} \epsilon_{\text{xx}}=\frac{\partial \text{u}}{\partial \text{x}}+\frac{\text{1}}{\text{2}}{\left(\frac{\partial \text{w}}{\partial \text{x}}\right)}^{\text{2}},\;\epsilon_{\text{yy}}=\frac{\partial \text{v}}{\partial \text{y}}+\frac{\text{1}}{\text{2}}{\left(\frac{\partial \text{w}}{\partial \text{y}}\right)}^{\text{2}},\;\gamma_{\text{xy}}=\frac{\partial \text{u}}{\partial \text{y}}+\frac{\partial \text{v}}{\partial \text{x}}+\frac{\partial \text{w}}{\partial \text{x}}\frac{\partial \text{w}}{\partial \text{y}} \end{array}$$ (4)

where u and v denote the in-plane displacement components, w represents the transverse deflection, $\epsilon_{xx}$ and $\epsilon_{yy}$ are the normal membrane strains, and $\gamma_{xy}$ is the in-plane engineering shear strain. The stress resultants and moment resultants follow from the linear elastic constitutive law through thickness integration:


$$\begin{array}{c} {\text{N}}_{\text{xx}}=\text{C}(\epsilon_{\text{xx}}+\nu \epsilon_{\text{yy}}),\;{\text{N}}_{\text{yy}}=\text{C}(\epsilon_{\text{yy}}+\nu \epsilon_{\text{xx}}),\;{\text{N}}_{\text{xy}}=\text{C}\frac{\text{1}-\nu }{\text{2}}\gamma_{\text{xy}} \end{array}$$ (5)

$$\begin{array}{c} {\text{M}}_{\text{xx}}=\text{D}(\kappa_{\text{xx}}+\nu \kappa_{\text{yy}}),\;{\text{M}}_{\text{yy}}=\text{D}(\kappa_{\text{yy}}+\nu \kappa_{\text{xx}}),\;{\text{M}}_{\text{xy}}=\text{D}\frac{\text{1}-\nu }{\text{2}}\kappa_{\text{xy}} \end{array}$$ (6)

Here $C\;=\;E\text{h}/(\text{1}\;-\;\nu^{\text{2}})$ and $D\;=\;E{\text{h}}^{\text{3}}/[\text{12}(\text{1}\;-\;\nu^{\text{2}})]$ denote the membrane and bending stiffness, respectively, with E being Young’s modulus, h the shell thickness, and ν Poisson’s ratio. The curvatures are $\kappa_{xx}\;=\;-\partial^{\text{2}}w/{\partial x}^{\text{2}}$, $\kappa_{yy}\;=\;-\partial^{\text{2}}w/{\partial y}^{\text{2}}$, and $\kappa_{xy}\;=\;-\partial^{\text{2}}w/\partial x\partial y$. The time-periodic in-plane tension field is explicitly incorporated through the modified stress resultants:


$$\begin{array}{c} N_{ij}\left(t\right)=N_{ij}^{\text{0}}\left[\text{1}+\gamma \text{sin}\left(\Omega t\right)\right] \end{array}$$ (7)

where $N_{ij}(t)$ is the time-varying in-plane stress resultant, $N_{ij}^{\text{0}}$ is the static pretension resultant, γ is the normalized tension-amplitude coefficient, Ω is the excitation frequency, and t is physical time. This treatment converts the external thermal-equivalent force field into a periodic modulation of system stiffness and nonlinear coupling intensity. After the time-dependent terms are introduced into the governing equations, large-deflection effects are retained through the higher-order geometric nonlinear terms.

The coupled governing equations of motion, derived through Hamilton’s principle applied to the total energy functional, take the explicit form:


$$\begin{array}{c} \rho \text{h}\frac{\partial^{\text{2}}\text{u}}{\partial {\text{t}}^{\text{2}}}=\frac{\partial {\text{N}}_{\text{xx}}}{\partial \text{x}}+\frac{\partial {\text{N}}_{\text{xy}}}{\partial \text{y}} \end{array}$$ (8)

$$\begin{array}{c} \rho \text{h}\frac{\partial^{\text{2}}\text{v}}{\partial {\text{t}}^{\text{2}}}=\frac{\partial {\text{N}}_{\text{xy}}}{\partial \text{x}}+\frac{\partial {\text{N}}_{\text{yy}}}{\partial \text{y}} \end{array}$$ (9)

$$\begin{array}{c} \rho \text{h}\frac{\partial^{\text{2}}\text{w}}{\partial {\text{t}}^{\text{2}}}+\text{c}\frac{\partial \text{w}}{\partial \text{t}}=\frac{\partial^{\text{2}}{\text{M}}_{\text{xx}}}{\partial {\text{x}}^{\text{2}}}+\text{2}\frac{\partial^{\text{2}}{\text{M}}_{\text{xy}}}{\partial \text{x}\partial \text{y}}+\frac{\partial^{\text{2}}{\text{M}}_{\text{yy}}}{\partial {\text{y}}^{\text{2}}}+{\text{L}}_{\text{nl}}(\text{w})+{\text{N}}_{\text{param}}\left(\text{w},\text{t}\right) \end{array}$$ (10)

where c is the viscous damping coefficient, $N_{xx}$, $N_{yy}$, and $N_{xy}$ are membrane stress resultants, and $M_{xx}$, $M_{yy}$, and $M_{xy}$ are bending moment resultants. The nonlinear geometric operator ${\text{L}}_{nl}$ and the parametric excitation operator $N_{param}$ are explicitly defined as:


$$\begin{array}{c} {\text{L}}_{\text{nl}}(\text{w})=\frac{\partial }{\partial \text{x}}({\text{N}}_{\text{xx}}\frac{\partial \text{w}}{\partial \text{x}}+{\text{N}}_{\text{xy}}\frac{\partial \text{w}}{\partial \text{y}})+\frac{\partial }{\partial \text{y}}({\text{N}}_{\text{xy}}\frac{\partial \text{w}}{\partial \text{x}}+{\text{N}}_{\text{yy}}\frac{\partial \text{w}}{\partial \text{y}}) \end{array}$$ (11)

$$\begin{array}{c} {\text{N}}_{\text{param}}(\text{w},\text{t})\;={\text{ N}}_{\text{xx}}\left(\text{t}\right)\frac{\partial^{\text{2}}\text{w}}{\partial {\text{x}}^{\text{2}}}+\text{2}{\text{N}}_{\text{xy}}\left(\text{t}\right)\frac{\partial^{\text{2}}\text{w}}{\partial \text{x}\partial \text{y}}+{\text{N}}_{\text{yy}}\left(\text{t}\right)\frac{\partial^{\text{2}}\text{w}}{\partial {\text{y}}^{\text{2}}} \end{array}$$ (12)

The boundary conditions for the fully clamped edges are prescribed as:


$$\begin{array}{c} \text{u }=\text{ v }=\text{ w }=\;\text{0},\;\frac{\partial \text{w}}{\partial \text{n}}\;=\;\text{0}\;\text{at}\;\text{x }=\;\text{0},\;{\text{L}}_{\text{x}}\;\text{and}\;\text{y }=\;\text{0},\;{\text{L}}_{\text{y}} \end{array}$$ (13)

where ∂/∂n denotes differentiation along the outward normal direction, $L_{x}$ and $L_{y}$ are the side lengths in the x and y directions, respectively. These continuous governing equations constitute the theoretical foundation from which the finite element spatial discretization is subsequently derived and upon which the multiple-scales perturbation analysis in Section 2.2 is constructed.

The governing equations are spatially discretized by the finite element method by dividing the thin shell into surface elements; the in-plane displacement and the lateral deflection serve as degrees of freedom, thereby yielding a discretized set of dynamic equations:


$$\begin{array}{c} 𝐌\ddot{𝐪}+𝐂\dot{𝐪}+𝐊\left(𝐪,t\right)𝐪=0 \end{array}$$ (14)

M is the mass matrix, C is the damping matrix, K(q,t) is the displacement-dependent stiffness matrix containing the time-varying tension contribution, and q is the global degree-of-freedom vector. The stiffness matrix is updated at each step according to both displacement nonlinearity and tension modulation, so that the dynamic response captures in-plane stretching, bending coupling, and parametric excitation consistently.

The boundary conditions are implemented by constraining the nodal displacement and rotational degrees of freedom, which preserves compatibility between global stiffness and local deflection. An implicit time-integration scheme is used for the governing equations, and the higher-order nonlinear terms are retained during each iteration. This approach balances numerical stability with accurate representation of the system dynamics.

The pretension and periodic-modulation parameters are summarized in Table 1. The static pretension sets the baseline stiffness level of the system, the modulation-amplitude coefficient determines the strength of parametric excitation, and the excitation frequency controls whether the response enters the resonance region. Frequency detuning measures the deviation from the natural-frequency combination condition, whereas the initial phase controls the timing of energy input. Together, these parameters determine the time-dependent modulation depth of the tension field and the sensitivity range of the dynamic response.

**Table 1:** Pretension and time-varying tension parameters.

Parameter	Value	Description
Static in-plane prestress	1200	Initial uniform membrane tension level
Modulation amplitude coefficient	0.18	Relative amplitude of periodic modulation
Excitation frequency	14.5	Frequency of periodic tension variation
Frequency detuning	0.35	Deviation from internal resonance condition
Initial phase	0	Phase angle of harmonic modulation

The parameters in Table 2 characterize structural damping and environmental perturbations. The mass- and stiffness-proportional damping coefficients determine the rate of energy dissipation and the relative dominance of amplitude growth or decay. Initial tension nonuniformity represents manufacturing or deployment defects in the internal stress field. The thermal-equivalent tension fluctuation quantifies the strength of orbital thermal cycling. The modulation period defines the excitation time scale and is therefore directly related to long-term integration stability and cumulative energy transfer.

**Table 2:** Damping and dynamic related parameters.

Parameter	Value	Description
Mass-proportional damping coefficient	2.00 × 10^−4^	Rayleigh damping mass term coefficient
Stiffness-proportional damping coefficient	3.50 × 10^−6^	Rayleigh damping stiffness term coefficient
Initial tension non-uniformity	1.5	Spatial deviation of in-plane tension
Thermal-equivalent tension fluctuation	85	Tension variation induced by thermal load
Modulation period	0.433	Period of harmonic tension variation

**Fig 2:**
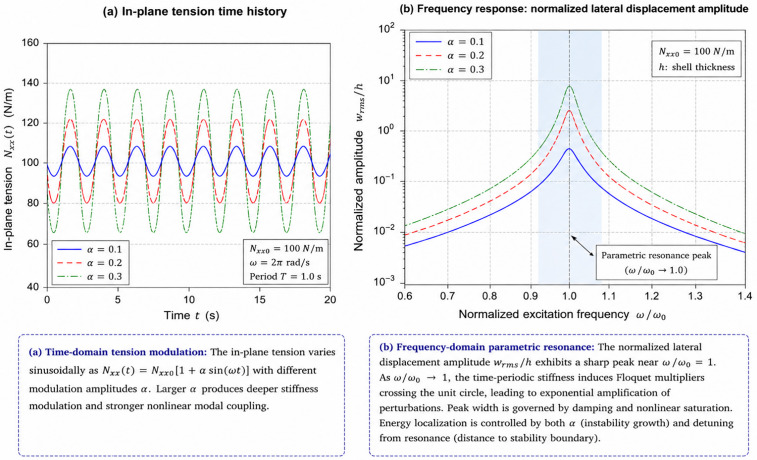
Time-domain and frequency-domain response characteristics of the parametrically excited thin-shell model. **(a)** In-plane tension time histories for normalized modulation-amplitude coefficients γ = 0.1, 0.2, 0.3 at static pretension $N^{\text{0}}\;=\text{ 100 }N/m$. **(b)** Normalized lateral displacement amplitude versus normalized excitation frequency, showing the parametric-resonance peak.

Fig 2 illustrates the dual nature of parametric excitation through time-domain tension modulation and frequency-domain resonance amplification. In Fig 2a, the sinusoidal tension variation directly modulates the system stiffness matrix through the geometric stiffness term in Eq. (12); larger γ produces deeper stiffness modulation and stronger nonlinear modal coupling. Fig 2b shows the corresponding frequency response. As the excitation frequency approaches the combined-resonance region, the normalized displacement exhibits a sharp peak followed by rapid decay after detuning, which is characteristic of parametric resonance. This behavior occurs because the time-periodic stiffness causes Floquet multipliers to cross the unit circle, rendering the trivial solution unstable and exponentially amplifying small perturbations. The peak width is governed by damping and nonlinear saturation, while the peak location confirms that energy localization is jointly controlled by tension-fluctuation intensity and frequency detuning.

### 2.2 Implementing multi-scale perturbation expansion to separate fast and slow dynamic processes

The method of multiple scales is applied directly to the continuous governing equations presented in Section 2.1. The displacement vector is expanded around the static equilibrium configuration. The fast time scale describes the carrier oscillations, whereas the slow time scale describes the evolution of the amplitude envelope.

Starting from the high-fidelity governing equations, a multiple-scales perturbation technique is introduced to separate high-frequency vibration from the slowly varying amplitude envelope and to reveal the nonlinear modal-coupling equations. Two time scales are defined: the fast scale $\tau_{\text{0}}\;=\;t$ and the slow scale $\tau_{\text{1}}\;=\;\epsilon t$, where t is physical time and ϵ is a dimensionless small parameter measuring the combined order of weak damping, detuning, and nonlinear stiffness. The displacement field is expanded to third order with respect to ϵ as follows:


$$\begin{array}{c} 𝐪\left(t\right)=\in {𝐪}_{\text{1}}\left(\tau_{\text{0}},\tau_{\text{1}}\right)+\in^{\text{2}}{𝐪}_{\text{2}}\left(\tau_{\text{0}},\tau_{\text{1}}\right)+\in^{\text{3}}{𝐪}_{\text{3}}\left(\tau_{\text{0}},\tau_{\text{1}}\right)+\text{O}\left(\in^{\text{4}}\right) \end{array}$$ (15)

Here, q(t) is the generalized displacement vector, $q_{\text{1}}$, $q_{\text{2}}$, and $q_{\text{3}}$ are the first-, second-, and third-order displacement corrections, and $\tau_{\text{0}}$ and $\tau_{\text{1}}$ denote the fast and slow time variables. Substituting the expansion into the thin-shell governing equations, the time derivatives are decomposed by the multiple-scales chain rule as follows:


$$\begin{array}{c} \frac{d}{dt}=\frac{\partial }{\partial \tau_{\text{0}}}+\in \frac{\partial }{\partial \tau_{\text{1}}} \end{array}$$ (16)

$$\begin{array}{c} \frac{d^{\text{2}}}{dt^{\text{2}}}=\frac{\partial^{\text{2}}}{\partial \tau_{\text{0}}^{\text{2}}}+\text{2}\in \frac{\partial^{\text{2}}}{\partial \tau_{\text{0}}\partial \tau_{\text{1}}}+\in^{\text{2}}\frac{\partial^{\text{2}}}{\partial \tau_{\text{1}}^{\text{2}}} \end{array}$$ (17)

The derivative operators in Eqs. (15) and (16) act on $\tau_{\text{0}}$ and $\tau_{\text{1}}$, respectively, ensuring the separation of high-frequency vibration from slowly varying amplitude evolution. After expansion of the nonlinear terms by order, the first-order problem gives the linear modal basis, whereas the second-order problem contains the resonant forcing terms that drive slow modal exchange [57,58]. The first-order free-vibration solution is written as:


$$\begin{array}{c} {𝐪}_{\text{1}}\left(\tau_{\text{0}},\tau_{\text{1}}\right)=𝐀\left(\tau_{\text{1}}\right)e^{i\omega \tau_{\text{0}}}+{𝐀}^{*}\left(\tau_{\text{1}}\right)e^{-i\omega \tau_{\text{0}}} \end{array}$$ (18)

where $A_{j}(\tau_{\text{1}})$ is the slowly varying complex amplitude of the j-th mode, $\omega_{j}$ is the corresponding natural frequency, and c.c. denotes the complex conjugate. At second order, products of modal functions, time-varying tension, and damping generate terms proportional to $exp(i\omega_{j}\tau_{\text{0}})$. If these terms are retained, they produce secular components that grow linearly with the fast time and violate the assumed asymptotic ordering. Therefore, the solvability condition is imposed by projecting the second-order residual onto each adjoint mode and setting the resonant component to zero. This harmonic-elimination step yields the second-order modulation equation:


$$\begin{array}{c} \frac{d𝐀}{d\tau_{\text{1}}}=𝐅\left(𝐀,{𝐀}^{*},\in ,t\right) \end{array}$$ (19)

The function $F_{j}$ contains the linear detuning term, viscous damping, parametric excitation, and nonlinear modal-coupling terms. The coupling coefficient between modes i, j, and k is obtained from the weighted overlap integral of their mode shapes with the geometric nonlinear operator. Consequently, a large value of the coefficient indicates strong spatial overlap of modal curvature and a more efficient pathway for energy transfer. In the third-order expansion, higher-order nonlinear corrections and slow-scale accumulation effects are retained, giving the complete amplitude-evolution system:


$$\begin{array}{c} \frac{d𝐀}{d\tau_{\text{1}}}={𝐅}_{\text{2}}\left(𝐀,{𝐀}^{*},\in \right)+\in {𝐅}_{\text{3}}\left(𝐀,{𝐀}^{*},\in \right) \end{array}$$ (20)

The second-order nonlinear contribution $F_{\text{2}}$ represents the leading resonant interaction, whereas the third-order correction $F_{\text{3}}$ accounts for nonlinear saturation and amplitude-dependent frequency shift. These terms are essential because the instability initially grows through parametric gain but eventually reaches a finite amplitude through nonlinear saturation. The modal energy on the slow scale is defined as follows:


$$\begin{array}{c} E_{m}\left(\tau_{\text{1}}\right)=\frac{\text{1}}{\text{2}}\left({\left|{\dot{𝐀}}_{m}\right|}^{\text{2}}+\omega_{m}^{\text{2}}{\left|{𝐀}_{m}\right|}^{\text{2}}\right) \end{array}$$ (21)

where $E_{j}$ is the energy of the j-th mode, $\omega_{j}$ is the natural frequency, and $dA_{j}/d\tau_{\text{1}}$ is the slow-scale amplitude derivative. This expression quantifies both intermodal energy-transfer efficiency and the degree of modal energy localization.

Through successive solution and secular-term elimination, the fast oscillation and slow envelope evolution are separated rigorously. The resulting modulation equations describe the energy-transport trajectory generated by nonlinear modal coupling and parametric excitation, while remaining consistent with the energy balance between parametric input, nonlinear redistribution, and damping.

The validity of the second-order multiple-scales approximation is restricted to a weakly nonlinear and slowly modulated regime in which damping, detuning, and the nonlinear frequency shift are all of order ε, and the transverse deflection remains small compared with the in-plane characteristic length even when it is large relative to the film thickness. Thus, the perturbation model is used here to predict resonance tongues, bifurcation direction, and threshold trends rather than to replace the full finite element integration under extreme thermal snapping or rapid attitude maneuvers. For strongly nonlinear events, the finite element platform in Section 2.3 updates the full displacement-dependent stiffness at every step, and the perturbation prediction is treated as a reduced-order diagnostic that is checked against the full-field response.

**Fig 3:**
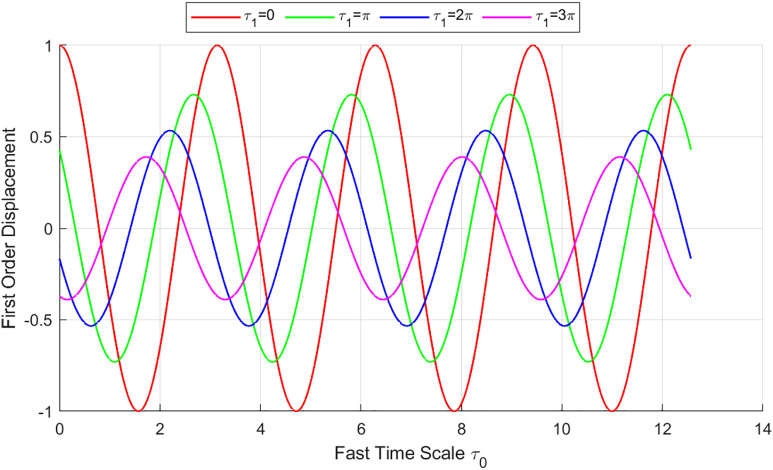
Fast-scale oscillations at different slow time points.

Fig 3 demonstrates fast-scale oscillations at several slow-time samples. The fast time scale is shown on the horizontal axis, and the first-order displacement is plotted on the vertical axis. Curves at different slow times have different amplitudes and phases, illustrating how the complex amplitude acts as the envelope of the fast oscillation. The gradual amplitude variation reflects energy redistribution caused by the combined effects of parametric stiffness modulation, geometric nonlinearity, and damping. The density of the oscillatory curves indicates the carrier-frequency content, whereas the envelope variation indicates the slow energy-transfer path. This separated oscillation pattern provides the basis for analyzing nonlinear dynamic responses of thin-film structures under parametric excitation.

### 2.3 Establishment of a high-fidelity finite element transient response simulation platform

Because analytical approaches are limited in describing global responses of strongly nonlinear and spatiotemporally coupled systems, a high-fidelity numerical simulation platform is constructed to reproduce the process from initial perturbation to steady-state pattern formation and to provide data for feature extraction. The thin-walled structure is discretized in the spatial domain by four-node curved shell elements. Each node contains two in-plane translational degrees of freedom and one transverse deflection degree of freedom. The element stiffness matrix consists of the linear elastic stiffness and the nonlinear geometric stiffness, leading to the global stiffness matrix:


$$\begin{array}{c} 𝐊\left(𝐪,t\right)=\sum_{e=\text{1}}^{N_{e}}{𝐁}_{e}^{T}{𝐃}_{e}{𝐁}_{e}+{𝐊}_{\text{NL},e}\left(𝐪\right) \end{array}$$ (22)

where $n_{e}$ is the total number of elements, $B_{e}$ is the element strain-displacement matrix, D is the material stiffness matrix, and $K_{e}^{NL}$ is the element nonlinear stiffness term. Time integration adopts the Newmark-β implicit scheme [59,60]. The displacement and velocity are iteratively updated with a step size Δt as follows:


$$\begin{array}{c} {𝐪}_{n+\text{1}}={𝐪}_{n}+\Delta t{\dot{𝐪}}_{n}+\Delta t^{\text{2}}\left[\left(\text{1}/\text{2}-\beta \right){\ddot{𝐪}}_{n}+\beta {\ddot{𝐪}}_{n+\text{1}}\right] \end{array}$$ (23)

$$\begin{array}{c} {\dot{𝐪}}_{n+\text{1}}={\dot{𝐪}}_{n}+\Delta t\left[\left(\text{1}-\delta \right){\ddot{𝐪}}_{n}+\delta {\ddot{𝐪}}_{n+\text{1}}\right] \end{array}$$ (24)

The displacement, velocity, and acceleration vectors at the n-th time step are denoted by $q_{n}$, $v_{n}$, and $a_{n}$, respectively, and β and δ are integration coefficients selected to ensure numerical stability during long-term integration. During iteration, Δt is adjusted by adaptive step-size control according to the acceleration variation rate, so that high-response and local-energy-concentration regions are captured accurately. The initial disturbance is introduced as low-amplitude white noise:


$$\begin{array}{c} 𝐪\left(t_{\text{0}}\right)={𝐪}_{\text{0}}+\eta ,\;\eta \sim \text{N}\left(\text{0},\sigma^{\text{2}}\right) \end{array}$$ (25)

where $q_{s}$ is the static equilibrium displacement, η is the random perturbation vector, and σ is the perturbation standard deviation. This initialization excites multimodal responses without prescribing a preferred pattern. Boundary conditions are imposed by constraining the relevant degrees of freedom, pinning selected nodes, or applying in-plane constraints to maintain compatibility between global stiffness and local displacement.

The simulation platform updates the nonlinear stiffness matrix and time-dependent parameters at each iteration, computes the global acceleration, and integrates the displacement and velocity to obtain a high-fidelity transient response. The time histories of all degrees of freedom are stored over the full domain, enabling analysis of modal energy distribution, nucleation and growth of localized high-amplitude spots, and subsequent spatiotemporal pattern evolution. The simulation parameters are shown in Table 3.

**Table 3:** Simulation parameters.

Parameter	Value	Description
Degrees of Freedom (per node)	3	Number of displacement components per node: 2 in-plane and 1 transverse
Initial Perturbation Standard Deviation (σ)	1e-4 m	Amplitude level of applied random noise
Random Perturbation Mean	0	Expected value of the initial disturbance
Total Simulation Duration	2000/ ω	Time span covering 2000 excitation cycles
Sampling Frequency	1000 Hz	Temporal resolution for data output
Spatial Grid Size (x-direction)	50	Number of discrete elements along the x-axis
Spatial Grid Size (y-direction)	50	Number of discrete elements along the y-axis
Minimum Adaptive Time Step	1e-6 s	Smallest time step allowed in integration
Maximum Adaptive Time Step	1e-4 s	Largest time step allowed in integration

To relate the idealized boundary model to deployable membranes mounted on flexible frames, the edge constraints can be generalized as translational and rotational spring supports. In this interpretation, the present clamped-edge results represent the high-frame-stiffness limit. If the support-frame natural frequencies are well separated from the membrane resonance tongue, frame flexibility mainly changes the effective detuning and produces only a small shift in the predicted γ and κ thresholds. If a frame mode falls within the combined-resonance bandwidth, however, the frame can act as an additional energy channel, lowering the localization threshold and biasing the pattern-selection criterion toward asymmetric or traveling states. Therefore, for low-frequency booms or highly compliant deployment frames, the same workflow should be applied to a coupled membrane-frame model rather than to an isolated membrane.

**Fig 4:**
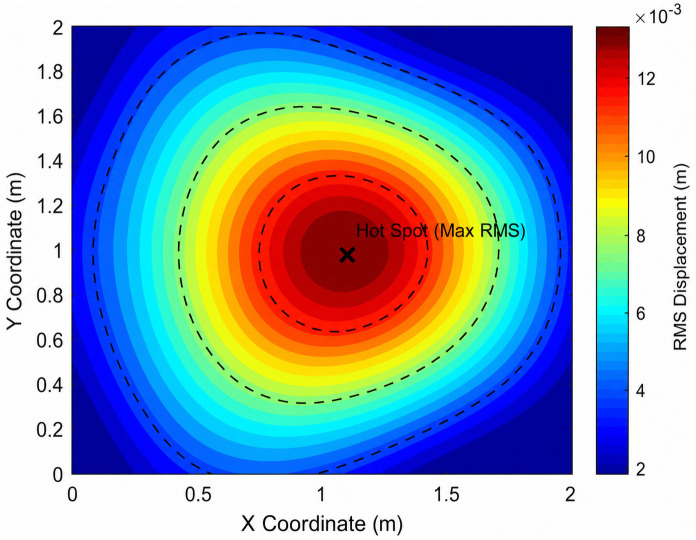
Spatial distribution of energy localization.

The RMS spatial distribution of the lateral displacement of the thin-film structure in the steady state under parametric excitation is shown in Fig 4. The figure is obtained from the displacement time histories of all nodes; the RMS value at each node is calculated from the corresponding time series. The results exhibit pronounced nonuniformity: an RMS hot spot appears near the center of the film with a value greater than 0.012 m, whereas the edge RMS is below 0.004 m, giving a center-to-edge ratio of approximately 3:1. The black dashed lines denote the 0.004 m, 0.008 m, and 0.012 m RMS contours. This visualization confirms that the simulation platform can capture parametric-excitation-induced energy localization, in which energy gradually concentrates from an initially broad distribution into an interior region with strong vibration. The hot spot provides the spatial seed for subsequent spatiotemporal pattern evolution.

### 2.4 Extracting spatiotemporal pattern features and quantifying modal energy distribution

After the full-field transient response is obtained, the localization region and pattern topology are extracted quantitatively by windowed spectral analysis, modal projection, and phase-space reconstruction. The full-field displacement sequence is sampled over space and time to form a spatiotemporal data matrix. A Fourier transform is then applied to convert the response into the frequency-wavenumber domain:


$$\begin{array}{c} \hat{w}\left(k_{x},k_{y},\omega \right)=\sum_{m=\text{0}}^{M-\text{1}}\sum_{n=\text{0}}^{N-\text{1}}\sum_{t=\text{0}}^{T-\text{1}}w\left(x_{m},y_{n},t\right)e^{-i\left(k_{x}x_{m}+k_{y}y_{n}-\omega t\right)} \end{array}$$ (26)

$w(k_{x},k_{y},\omega )$ represents the transformed complex amplitude, $w(x_{m},y_{n},t)$ represents the displacement record at spatial node $\left(x_{m},y_{n}\right)$ and time t, $k_{x}$ and $k_{y}$ are the spatial wavenumber components, and M, N, and T are the numbers of spatial and temporal sampling points. The extracted dominant frequency components after transformation are analyzed in the spatial domain to identify vibration modes and local amplification regions.

The full-field displacement vector is projected onto the modes to obtain the time histories of the modal participation factors for each order. The calculation expressions are as follows:


$$\begin{array}{c} q_{m}\left(t\right)=\phi_{m}^{T}𝐪\left(t\right) \end{array}$$ (27)

$q_{m}(t)$ is the m-th mode participation factor, $\phi_{m}$ is the m-th mode vector, and q(t) is the global degree-of-freedom vector. Modal energy is defined by the participation factor as:


$$\begin{array}{c} E_{m}\left(t\right)=\frac{\text{1}}{\text{2}}\left({\dot{q}}_{m}^{\text{2}}+\omega_{m}^{\text{2}}q_{m}^{\text{2}}\right) \end{array}$$ (28)

$E_{m}(t)$ is the energy of the m-th mode, and $q_{m}^{\cdot }$ denotes the time derivative of the modal participation factor. The modal energy proportion is used for calculation:


$$\begin{array}{c} \eta_{m}\left(t\right)=\frac{E_{m}\left(t\right)}{\sum_{j=\text{1}}^{N_{m}}E_{j}\left(t\right)} \end{array}$$ (29)

$\eta_{m}(t)$ is the energy proportion of the m-th mode, and $N_{m}$ is the total number of retained modes. This ratio quantifies whether energy is concentrated in a limited set of modes and, together with spatial RMS maps, whether it is concentrated in a specific region.

Phase space reconstruction maps the displacement-time series into a multi-dimensional space by constructing a delay coordinate vector, forming a phase trajectory, expressed as:


$$\begin{array}{c} 𝐗\left(t\right)=\left[w\left(t\right),w\left(t+\tau \right),w\left(t+\text{2}\tau \right),\ldots ,w\left(t+\left(d-\text{1}\right)\tau \right)\right] \end{array}$$ (30)

where X(t) is the reconstructed phase-space vector, τ is the time delay, and d is the embedding dimension. Phase-space trajectories are used to identify local amplitude enhancement and vibration-structure characteristics, and to distinguish stationary breathers from traveling waves. By combining Fourier transform, modal projection, and phase-space reconstruction, a complete spatiotemporal pattern characterization and modal-energy quantification procedure is established for the subsequent analysis of localization.

**Fig 5:**
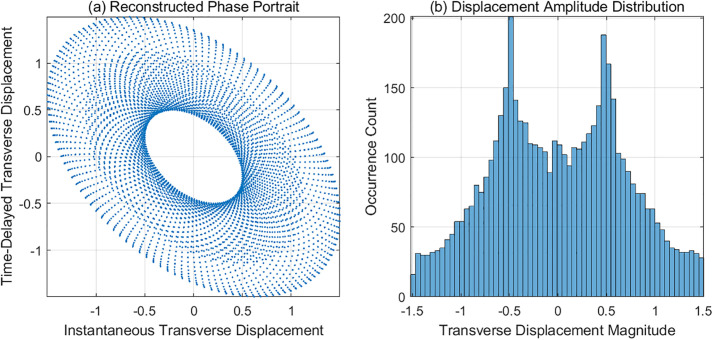
Phase space and amplitude statistics.

The two-dimensional phase diagram reconstructed from delayed coordinates is shown in Fig 5a. The x-axis represents the instantaneous transverse displacement, and the y-axis represents the time-delayed transverse displacement. The trajectory forms a closed orbit that periodically expands and contracts, indicating the presence of slow envelope modulation. The absence of scattered diffuse points suggests deterministic dynamics rather than random vibration. The periodic change in orbit radius corresponds to the amplitude-modulation frequency and represents cyclic energy redistribution inside the system. Fig 5b shows the statistical distribution of transverse displacement magnitude. The bimodal distribution indicates alternating high- and low-amplitude states, which arise from periodic thickening and thinning of the vibration envelope. The phase-plane loop and amplitude statistics together show that the vibration remains confined within a finite attraction region rather than diffusing throughout the entire modal space.

## 3. Data verification of energy localization and pattern evolution laws

### 3.1 Numerical data, ground-validation analogy, and setup

The numerical experiments in this study are based on the high-fidelity finite element transient-response platform. A square thin-film model is discretized by a 50 × 50 mesh of four-node curved shell elements. Each node has two in-plane translational degrees of freedom and one out-of-plane deflection degree of freedom, resulting in 7,500 degrees of freedom. The time-integration solver uses the implicit Newmark scheme with adaptive step-size control. The step size is constrained between 10^−6^ s and 10^−4^ s to capture local energy accumulation caused by abrupt variations in nonlinear stiffness. The total simulation duration covers 2000 excitation cycles, and the output sampling frequency is 1000 Hz, which is sufficient to record the spatiotemporal evolution details.

The initial condition is a zero-mean Gaussian white-noise perturbation with a standard deviation of 10^−4^ m, which excites multimodal responses and mimics weak random disturbances in the space microenvironment. Edge-node displacements are constrained to remove rigid-body motion and maintain global-stiffness consistency. The main control variables are the tension fluctuation amplitude coefficient and the excitation-frequency detuning. The former controls the strength of parametric resonance, whereas the latter adjusts the modal-coupling window. Rayleigh damping is adopted, with an equivalent modal damping ratio of approximately 0.001 to represent low dissipation in vacuum. In addition, an initial tension nonuniformity of 1.5% and thermal-equivalent tension fluctuations are introduced to represent the combined influence of orbital thermal cycling and fabrication imperfections on the stress field.

Data acquisition includes the transient displacement histories of all nodes. Post-processing yields the lateral-displacement RMS distribution, modal participation factors, phase-space trajectories, and frequency-wavenumber spectra. The dominant wavenumber and frequency components are then used to measure pattern spatial period, propagation velocity, and localization intensity, thereby establishing a continuous evidence chain from microscopic parameter modulation to macroscopic spatiotemporal ordering.

The present validation should be interpreted as a controlled numerical-experiment matrix rather than a direct in-orbit experiment. Nevertheless, the excitation model is constructed as an equivalent modal representation of distributed multiaxial parametric loading produced by thermal snapping, solar-pressure variation, and reaction-wheel disturbances. In a laboratory configuration, this condition can be approximated by orthogonal edge actuators or distributed piezoelectric patches whose phases and amplitudes are tuned so that the modal generalized forces and the frequency content match the target on-orbit spectrum. For simultaneous multiaxial inputs, the tension field can be expanded as a sum of harmonic components; only components whose combination frequencies fall inside the resonance bandwidth contribute strongly to localization. This equivalence criterion provides a practical route for future experimental validation while avoiding the overstatement that the current numerical matrix fully reproduces all space-environment loads.

### 3.2 Nonlinear stiffness surface and spatial evolution of lateral displacement amplitude

To verify the correctness of the functional relationship between nonlinear stiffness, displacement, and nonlinear coefficients, grid data was generated within the complete range of displacement from its negative maximum to its positive maximum, based on the mathematical expression of the squared term of the lateral deflection gradient in geometric nonlinear theory. The nonlinear coefficients covered a continuous range from weak to strong nonlinearity. The nonlinear stiffness value corresponding to each grid point was calculated based on a quadratic function relationship. A surface plotting method was used to ensure that the color was entirely determined by the numerical value, and a color spectrum was used to present the continuous change of stiffness value from low to high.

**Fig 6:**
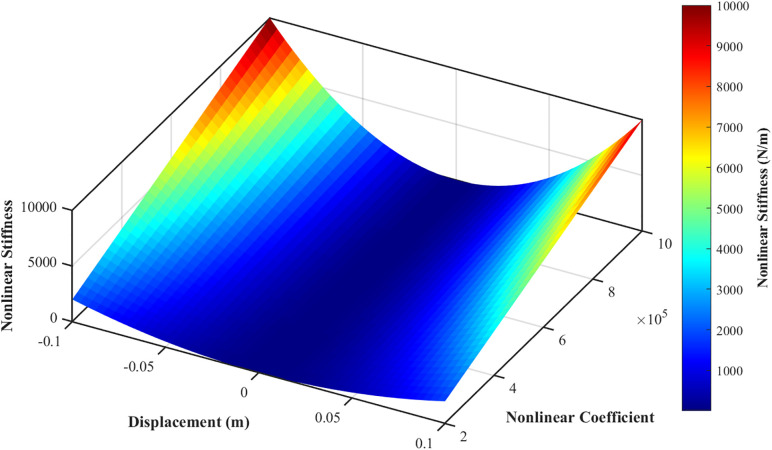
Nonlinear stiffness surface.

Fig 6 illustrates the functional relationship among nonlinear stiffness, displacement, and nonlinear coefficient. The displacement axis ranges from −0.1 m to 0.1 m, and the nonlinear coefficient ranges from 2 × 10^5^ N/m³ to 1 × 10⁶ N/m³. The color scale maps the nonlinear stiffness value, with blue indicating low stiffness and red indicating high stiffness. The surface has a parabolic shape along the displacement axis: nonlinear stiffness is minimal at zero displacement and increases quadratically with displacement amplitude. Along the nonlinear-coefficient axis, the surface increases approximately linearly, indicating that a larger nonlinear coefficient produces greater stiffness at the same displacement. This displacement-dependent stiffness is the source of nonlinear behavior in the dynamic equations and explains why the stiffness matrix changes with vibration state under parametric excitation.

**Fig 7:**
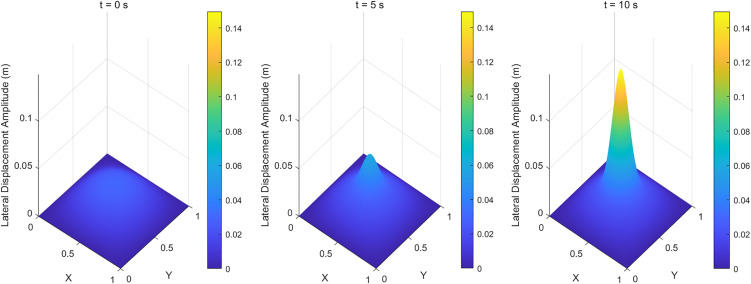
Spatial evolution of lateral displacement amplitude.

Fig 7 shows the temporal evolution of lateral displacement amplitude at each grid point. The response initially has a smooth low-order spatial distribution with a small center-to-boundary gradient, indicating that energy is distributed broadly across the domain. At the intermediate stage (t = 5 s), the central amplitude grows substantially, the local surface curvature increases, and the background amplitude remains low. This indicates that periodic in-plane tension modulation changes the modal equilibrium configuration and enhances nonlinear intermodal coupling. At the final stage, the central peak continues to grow while the localized region maintains a nearly fixed spatial width. The spatial pattern evolves from a global mode to a localized wave-packet structure, demonstrating cumulative energy feeding and redistribution through the interaction of geometric nonlinearity and parametric excitation. Because the plotted amplitude is nonnegative, it emphasizes vibration magnitude rather than phase; nevertheless, the comparison across time clearly shows that the dynamic response has left the linear-superposition regime and entered a spatially nonuniform vibration state.

### 3.3 Numerical solution and phase space reconstruction of amplitude evolution

The numerical solution is based on the second-order slow-amplitude modulation equation. The complex amplitude is treated as a slow-time variable, and nonlinear growth and saturation are represented by van der Pol-type terms. Integration is performed over the slow-time domain with a small step size, and the amplitude and its derivative are updated point by point. The complete amplitude path is followed from a small initial perturbation. The one-dimensional time series is then reconstructed into a two-dimensional phase trajectory, which reveals whether a closed-loop attractor is formed after sufficiently long evolution.

**Fig 8:**
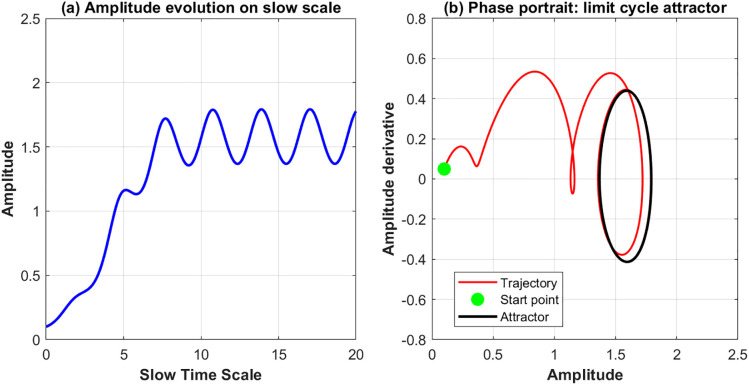
Slow-scale amplitude dynamics and phase space characteristics.

Fig 8 demonstrates the slow-scale amplitude evolution and corresponding phase-space characteristics under parametric excitation. In Fig 8a, the amplitude grows from a small initial value and then evolves into periodic fluctuation. This behavior results from competition between the linear growth term and nonlinear saturation in the modulation equation. The parametric excitation continues to modulate the response after amplitude saturation, producing oscillation around the dynamic equilibrium. In Fig 8b, the trajectory in the amplitude-derivative plane spirals outward and converges to a closed loop. This closed loop represents a limit-cycle attractor, showing that the transient response converges to a stable periodic oscillatory mode. The convergence confirms the presence of a stationary breather state: energy periodically exchanges between modes but has no net spatial flux, which is a key signature of nonlinear energy localization under parametric excitation.

### 3.4 Energy accumulation criterion for combined resonance points near the excitation frequency

Parametric excitation frequency sweeps are performed around two low-order bending modes. The first and second natural frequencies are set to ω_1_ = 0.95 ω_0_ and ω_2_ = 1.05 ω_0_, where ω_0_ is the reference fundamental frequency used for normalization. According to nonlinear dynamics theory, strong modal coupling occurs when the excitation frequency is close to half the sum of the two modal frequencies, Ω=(ω_1_ + ω_2_)/2. In the evaluation, the excitation amplitude is fixed and the excitation frequency is swept around the combined-resonance region. At each frequency point, the ratio of the maximum local amplitude to the global RMS amplitude is calculated. A ratio greater than 3.0 is used as the diagnostic criterion for pronounced localization. This procedure quantifies whether combined resonance is the dominant mechanism for energy accumulation.

**Fig 9:**
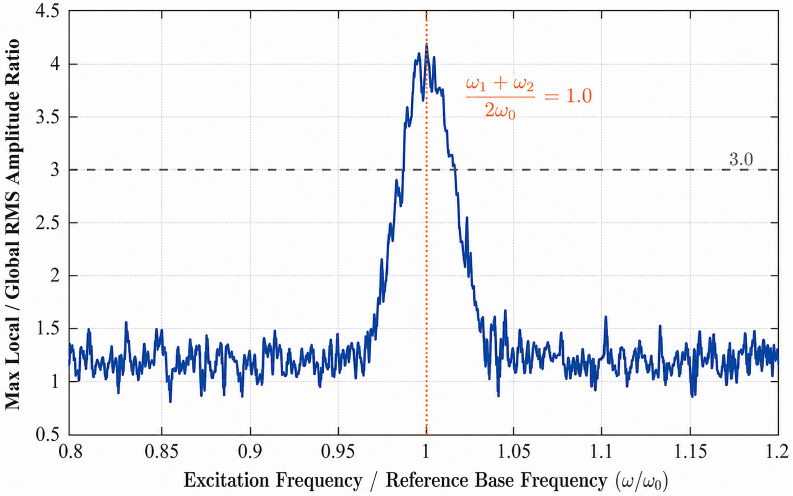
Energy localization response induced by combined resonance.

Fig 9 shows the ratio of maximum local amplitude to global RMS amplitude during a normalized excitation-frequency scan. The x-axis is the excitation frequency normalized by the reference frequency, and the y-axis is the energy-concentration ratio. A pronounced peak appears near the normalized frequency of 1.0, corresponding to the combined resonance condition. At this point, energy is transferred abruptly from the global vibration field into a much stronger local oscillation region. The frequency band in which the ratio exceeds 3.0 defines the localization band, indicating a region of modal instability in which the original uniform vibration mode is broken. The off-resonance background reflects weakly coupled energy spreading. Damping and nonlinear restoring stiffness jointly determine the bandwidth of the resonance peak, and the agreement between the numerical peak and the theoretical combined-resonance line verifies the mode-interaction mechanism of energy accumulation.

Physically, the γ > 0.20 and Amax/ARMS>3.0 thresholds should be interpreted as operational localization criteria rather than universal constants. The tension threshold marks the point at which parametric energy input over one modulation period exceeds the combined effect of damping and nonlinear redistribution, so that the relevant Floquet multiplier becomes larger than unity. The amplitude-ratio threshold is a measurable response indicator that separates weak spatial modulation from damaging local vibration hot spots. Consequently, the threshold values depend on damping, detuning, support stiffness, and manufacturing imperfections, whereas the underlying mechanism is the same: instability of a coupled modal pair followed by nonlinear saturation.

### 3.5 Influence of nonlinear modal coupling strength on pattern type

This assessment examines topological changes of spatiotemporal patterns induced by nonlinear modal coupling strength. The complex-amplitude modulation equations obtained from multiple-scales perturbation are used to compute the instantaneous energy time series of the dominant and secondary modes. An intermodal energy-exchange rate is introduced to quantify the transfer rate from the dominant mode to the secondary mode, normalized by the initial dominant-mode energy. A series of high-precision transient simulations is then performed at the combined resonance frequency while varying only the tension-fluctuation amplitude. For each parameter set, modal projection and energy-flow analysis are carried out during long-time evolution. The mean coupling intensity in the steady regime is statistically analyzed, and the corresponding pattern type is identified as a stationary breather or traveling pattern through phase-space reconstruction and frequency-wavenumber spectrum analysis. This procedure establishes a quantitative map between coupling strength and pattern topology.

**Fig 10:**
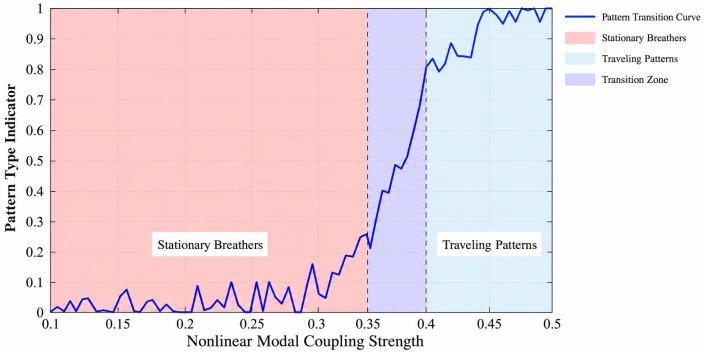
Coupling strength determines the choice of spatiotemporal mode.

Fig 10 presents the relationship between nonlinear modal coupling strength and spatiotemporal pattern topology. The horizontal axis is the nonlinear modal coupling strength, and the vertical axis is a pattern indicator measuring the degree of spatiotemporal symmetry breaking. For κ < 0.35, the indicator remains low and the response corresponds to stationary breathers, in which energy cyclically focuses and defocuses within a fixed localized region. Phase locking maintains an overall stationary envelope. For κ > 0.40, the indicator increases sharply, showing a transition to traveling patterns in which a unidirectional energy envelope moves across the film surface. This motion is induced by continuous asymmetric energy transport among modes and breaks spatial translational symmetry. The interval 0.35 ≤ κ ≤ 0.40 is a transitional region associated with multistability and chaotic precursors. The threshold is determined by competition between nonlinear dispersion and dissipation: stronger coupling facilitates modal phase slip, which destabilizes standing patterns and generates traveling waves. The result confirms that topology is selected indirectly through modal coupling rather than directly by excitation amplitude.

A practical distinction between true traveling waves and slowly drifting standing waves is made using three diagnostics. First, a true traveling pattern has a persistent nonzero phase velocity obtained from the slope of the unwrapped phase in the x-t plane, whereas a drifting standing wave shows intermittent node motion without a stable phase gradient. Second, the energy-flux density has a nonzero time-averaged sign for a traveling pattern but averages to nearly zero for a standing wave with boundary-induced drift. Third, the frequency-wavenumber spectrum of a traveling pattern is asymmetric in the ± k components, whereas a drifting standing wave retains nearly symmetric sidebands. These criteria reduce the risk of misclassifying patterns caused by slight boundary asymmetry or nonuniform pretension.

### 3.6 Sensitivity analysis of initial perturbation amplitude to localization onset time

This assessment analyzes the sensitivity of localization onset time to the initial perturbation amplitude. Under fixed system parameters, with the excitation frequency at combined resonance and the tension-fluctuation amplitude fixed, four numerical experiments are performed. Zero-mean Gaussian white noise is added to the initial displacement field with standard deviations of 10^−5^, 10^−4^, 10^−3^, and 10^−2^ m. Each experiment uses the same grid and time step, and the local-to-global RMS ratio is monitored in real time. The localization onset time is defined as the time at which this ratio first reaches 3.0. Multiple realizations are used to reduce randomness, and the mean and standard deviation of onset time are recorded. The long-term pattern morphology is also monitored to verify whether the final attractor is independent of the initial condition.

Table 4 quantifies the dependence of localization onset time on initial perturbation amplitude. As the perturbation amplitude increases from 10^−5^ to 10^−2^ m, the localization onset time decreases from 862 to 187 excitation cycles; each order-of-magnitude increase reduces the onset time by approximately 40%. This trend reflects exponential sensitivity during the early stage of modulation instability: a smaller perturbation requires a longer nonlinear amplification process before reaching the localization threshold. The standard deviation decreases as perturbation amplitude increases, suggesting that stronger perturbations drive the system more rapidly away from stochastic initial conditions and toward a deterministic evolution route. The final pattern type remains unchanged in all cases, indicating that the long-term dynamics is governed by the attractor created by parametric excitation and structural nonlinearity rather than by the initial disturbance amplitude.

**Table 4:** Influence of initial perturbation amplitude on energy localization onset time.

Initial Perturbation Amplitude	Localization Onset Time (Excitation Cycles)	Time Reduction Ratio (%)	Steady-State Pattern Type
1.0 × 10^−5^	862 ± 28	—	Traveling Pattern
1.0 × 10^−4^	523 ± 19	39.3	Traveling Pattern
1.0 × 10^−3^	312 ± 12	40.3	Traveling Pattern
1.0 × 10^−2^	187 ± 8	40.1	Traveling Pattern

### 3.7 Quantitative correlation between tension fluctuation amplitude and local energy peak

The evaluation procedure establishes a quantitative mapping between tension-fluctuation amplitude and the steady local energy peak. The excitation frequency is fixed at the combined resonance point to keep the modal-coupling condition constant. The tension fluctuation amplitude, normalized by the static tension, is sampled from 0.05 to 0.30. For each parameter set, a high-accuracy transient response simulation is performed. After the system reaches steady state, the maximum local amplitude of the full-field transverse displacement is extracted as a measure of energy concentration. Each condition is repeated three times, and the mean and standard deviation of the steady maximum local amplitude are calculated. This procedure isolates the regulatory effect of excitation amplitude on nonlinear energy focusing and provides a critical design threshold for disturbance resistance.

**Fig 11:**
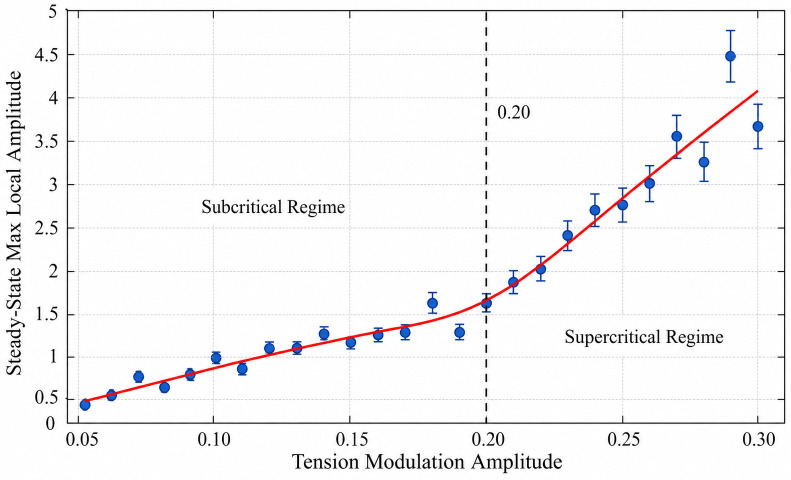
Nonlinear response of tension modulation amplitude-local amplitude.

Fig 11 shows the nonlinear relationship between tension-fluctuation amplitude and steady maximum local amplitude. The x-axis is the normalized tension-fluctuation amplitude, and the y-axis is the maximum local amplitude. When the modulation amplitude is below 0.20 of the static tension, the response increases slowly, indicating a subcritical regime in which nonlinear stiffness effects remain limited. Once the modulation amplitude exceeds 0.20, the slope increases sharply and the local amplitude grows rapidly, indicating entry into the supercritical regime. This transition occurs because the parametric excitation crosses the modal-instability threshold, leading to strong nonlinear modal coupling and positive feedback in energy focusing. The error bars increase at higher amplitudes because the system becomes more sensitive to initial disturbances and discretization. The threshold location is controlled by geometric nonlinearity, damping, detuning, and boundary stiffness, and it provides a quantitative basis for on-orbit tension-control design.

Table 5 connects the uncertainty analysis with the localization threshold discussed in Section 3.7. It shows that the nominal tension-fluctuation threshold reported for the ideal membrane should not be interpreted as a universal material constant, but rather as a baseline value for the modeled configuration. Initial waviness mainly shortens the onset time by seeding resonant modal pairs; residual stress shifts the effective detuning and can either increase or decrease the required modulation amplitude; residual curvature breaks spatial symmetry and promotes slow drift; and thickness variation creates local stiffness and mass deviations that favor hot-spot nucleation. Therefore, the threshold used in design should be recalibrated using measured deployment shapes, residual-stress maps, and thickness-tolerance data before applying the localization criterion to a real membrane.

**Table 5:** Sensitivity pathways affecting the tension-fluctuation localization threshold.

Uncertainty source	Model entry	Expected effect on localization threshold	Design implication
Initial geometric imperfection	Initial deflection field w0(x,y)	Seeds the resonant modal pair and usually advances localization onset; large imperfections can slightly lower the apparent γc.	Include measured deployment shape in the initial state and keep local waviness below the allowable RMS tolerance.
Residual folding/deployment stress	Static and spatially varying pretension N0 + ΔN(x,y)	Shifts the natural frequencies and can either raise or lower γc depending on whether the residual stress moves the system away from or toward combined resonance.	Measure residual stress after deployment and retune the excitation-detuning margin.
Residual bending curvature	Bias curvature in the geometric stiffness term	Breaks symmetry and may convert a stationary breather into a slowly drifting or traveling pattern.	Use symmetric packaging and post-deployment flattening where possible.
Thickness variation	Local bending stiffness D ∝ h³ and areal mass ρh	Reduces local stiffness in thinner regions, making them preferred hot-spot nucleation sites and lowering local γc.	Apply thickness-quality control and place damping/stiffening elements near statistically weak zones.

### 3.8 The constraint effect of spatial scale on spatial period of patterns

This evaluation examines how structural geometry constrains the spatial features of self-organized patterns. Square thin-film models with side lengths of 1, 2, 3, and 4 m are considered with identical material properties, boundary conditions, static pretension, and excitation-frequency settings. High-fidelity transient simulations are conducted for each size. After the system reaches steady state, the dominant wavenumber is extracted by a two-dimensional spatial Fourier transform, and the basic pattern period is calculated. The spatial period is then divided by the side length to obtain a dimensionless scaling factor. Three independent runs are performed for each case, and the mean and standard deviation of the spatial period and scaling factor are analyzed statistically. This procedure tests whether the pattern has an intrinsic length scale determined by nonlinear dispersion and excitation wavenumber rather than by absolute structural size alone.

Table 6 shows the scaling law between thin-film geometry and steady-state pattern spatial period. As the side length increases from 1 to 4 m, the pattern period increases nearly linearly from 0.281 to 1.124 m, with the scaling factor converging to approximately 0.28. This fixed ratio suggests that the pattern wavelength is determined by local dynamic equilibrium rather than by the absolute macroscopic size. The wavelength is selected by the nonlinear dispersion relation and dominant parametric-excitation wavenumber under normalized coordinates. Boundary conditions determine modal node positions, whereas nonlinear stiffness, inertia, and periodic stiffness modulation determine the self-organized wavelength. Small variations in the scaling factor arise from discretization and edge effects. The result supports the use of scaled models for square or near-square deployable membranes, provided the nondimensional excitation and boundary parameters are preserved.

**Table 6:** Scale relationship between pattern spatial period and structural dimensions.

Membrane Side Length (m)	Pattern Spatial Period (m)	Proportionality Coefficient (Period/ Side Length)
1	0.281 ± 0.006	0.281 ± 0.006
2	0.563 ± 0.009	0.282 ± 0.005
3	0.842 ± 0.012	0.281 ± 0.004
4	1.124 ± 0.015	0.281 ± 0.004

The proportionality λ/L ≈ 0.28 should not be extrapolated without modification to highly anisotropic membranes, such as strip-like sails with kilometer-scale length and meter-scale width. For aspect ratios close to unity, the selected wavenumbers in the two in-plane directions remain comparable and a single characteristic length L is meaningful. For highly anisotropic geometries, the dominant pattern is governed by the vector wavenumber components and usually locks to the shorter dimension or to the direction with stronger pretension gradients. In such cases, separate ratios λx/Lx and λy/Ly, together with anisotropic membrane stiffness and boundary compliance, must be used. Therefore, the reported scale invariance is a validated result for square and near-square membranes and a design hypothesis requiring additional correction for extreme aspect ratios.

### 3.9 Energy dissipation verification of pattern stability during long-term evolution

This evaluation verifies the dynamic stability of spatiotemporal patterns under parametric excitation in a weakly dissipative medium. Rayleigh damping is introduced into the governing equations, with a modal damping ratio of 0.001 to represent the low material energy dissipation of space films. A stationary breather and a traveling pattern are selected as representative steady states, and transient integration is continued for 2000 excitation cycles. The amplitude envelope of the local high-amplitude region is monitored, and the relative decay rate with respect to the initial steady state is calculated. A pattern is considered stable over the simulated horizon if the amplitude decay remains below 5%. This procedure removes transient-growth interference and verifies that the ordered structures observed in the simulations are not intermediate states but attractors sustained by the balance between constant parametric input and weak dissipation.

**Fig 12:**
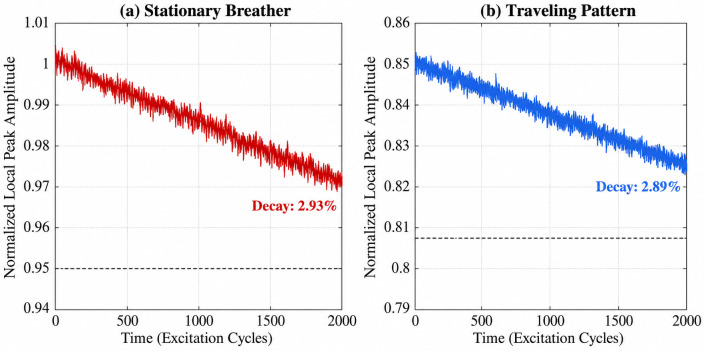
Long-term amplitude stability under weak damping.

Fig 12 confirms the long-term dynamic stability of the spatiotemporal patterns over the simulated 2000-cycle horizon. The horizontal axis is the number of excitation periods, and the vertical axis is the normalized local peak amplitude. In Fig 12a, the stationary breather amplitude decreases from 1.000 to approximately 0.97, corresponding to a decay rate of 2.93%. In Fig 12b, the traveling pattern decreases from 0.85 to approximately 0.825, with a decay rate of 2.89%. The gray dashed line indicates the 5% stability threshold, and all points remain above this line. The small amplitude oscillations reflect dynamic equilibrium between parametric energy input and weak damping loss. Thus, both patterns behave as persistent attractors within the verified numerical horizon.

Extrapolation from 2000 cycles to millions of on-orbit cycles requires an averaged slow-flow argument rather than direct numerical continuation alone. If the fixed point or limit cycle of the modulation equations has negative transverse Floquet exponents and the mission parameters remain inside the same attraction basin, the localized pattern can persist over much longer times. However, slow mechanisms may eventually destabilize it, including gradual thermal drift of natural frequencies, creep or stress relaxation, frequency-dependent damping, material memory, radiation-induced property changes, and support-frame aging. Therefore, the 2000-cycle result should be interpreted as evidence of attractor persistence over the simulated horizon, while mission-level certification should include parameter-drift monitoring and periodic retuning of pretension or active damping.

### 3.10 Multi-stage spatiotemporal evolution analysis of energy localization

**Fig 13:**
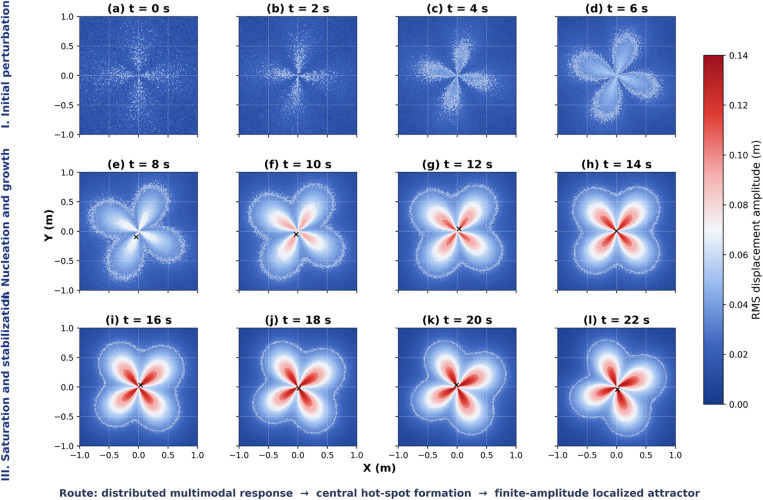
Spatiotemporal evolution of energy localization patterns: 12 time snapshots.

To comprehensively characterize the complete evolution process from initial perturbation to steady-state energy localization, we conducted systematic numerical experiments capturing 12 distinct time snapshots. The simulation setup maintains consistent parametric excitation conditions (combined resonance frequency Ω=(ω_1_ + ω_2_)/2, tension fluctuation amplitude γ = 0.22) while monitoring the spatiotemporal evolution over 2000 excitation cycles. The 12-grid visualization presented in Fig 13 reveals the progressive energy concentration mechanism.

Fig 13 presents the 12-stage spatiotemporal evolution of energy-localization patterns from initial uniform perturbation (t = 0 s) to fully developed steady-state localization (t = 22 s). The visualization reveals three phases. First, during the initial perturbation phase (t = 0–4 s), the system exhibits nearly uniform low-amplitude oscillations with weak spatial variation, indicating that energy is distributed across multiple modes. Second, during the nucleation and growth phase (t = 6–14 s), a pronounced energy hot spot forms near the structural center, and the RMS displacement amplitude increases substantially relative to the initial state. The localized region develops a clear boundary and a characteristic wavelength consistent with the scale relationship identified in Section 3.8. Third, during the saturation and stabilization phase (t = 16–22 s), the energy localization reaches a dynamic equilibrium in which parametric input is balanced by damping and nonlinear redistribution. This temporal sequence directly visualizes the route from homogeneous response to localized spatiotemporal ordering.

### 3.11 Pattern topology selection under varying modal coupling strengths

**Fig 14:**
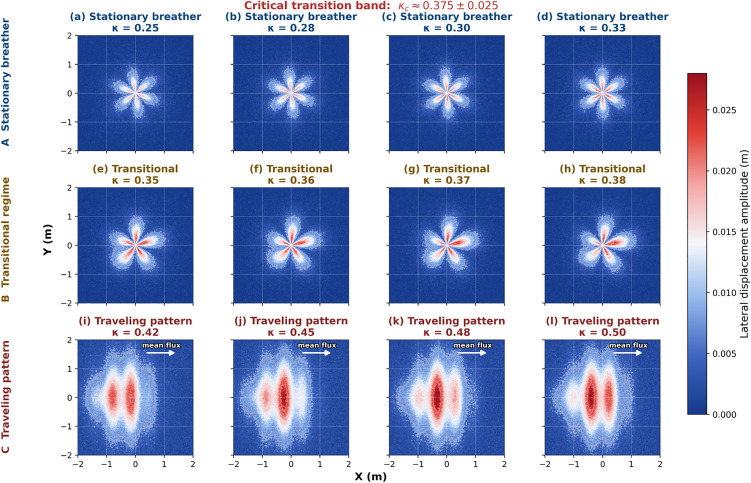
Pattern-type selection under different nonlinear modal-coupling strengths: 12 cases.

The nonlinear modal coupling strength serves as the fundamental control parameter governing pattern type selection. To establish the quantitative mapping between coupling intensity and pattern topology, we designed 12 comparative simulations spanning the coupling strength range κ∈[0.25, 0.50]. Each case maintains identical excitation frequency at the combined resonance point while systematically varying the tension modulation amplitude to achieve the target coupling strength. The resulting pattern morphologies are categorized and visualized in Fig 14.

Fig 14 demonstrates the pattern-selection mechanism across 12 nonlinear modal-coupling strengths. The results reveal three regimes. (A) Stationary breather regime (κ < 0.35): at low coupling strength, the system evolves into localized stationary breathers characterized by phase-locked energy oscillation within a fixed spatial region, nearly circular amplitude contours, and zero mean energy flux across the localization boundary. (B) Transitional regime (0.35 ≤ κ ≤ 0.38): the system exhibits multistability, intermittent switching, and weakly propagating states. The patterns show broken spatial symmetry and sporadic redistribution events. (C) Traveling-pattern regime (κ > 0.40): the system undergoes spontaneous symmetry breaking and forms traveling localized envelopes with nonzero mean phase velocity and directional energy flux. These results confirm that κ is the immediate topological control parameter, whereas the excitation amplitude acts indirectly by changing κ through the modal-coupling pathway.

### 3.12 Frequency-wavenumber spectrum analysis for modal energy distribution

To rigorously quantify the modal energy distribution and validate the combined resonance mechanism, we performed comprehensive frequency-wavenumber spectrum analysis under varying excitation conditions. The 12-grid simulation matrix comprises two parameter sweeps: (1) frequency sweep around the combined resonance point (Ω/ω_0_ = 0.85–1.20), and (2) tension fluctuation amplitude sweep (γ = 0.10–0.25). Two-dimensional spatial Fourier transforms were computed for each steady-state response to extract the dominant wavenumber components and their spectral power distribution.

**Fig 15:**
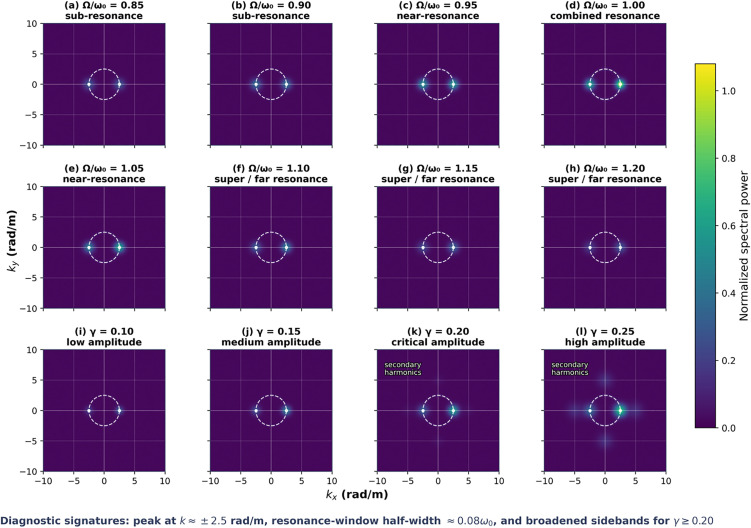
Frequency-wavenumber spectrum analysis under different excitation conditions: 12 cases.

Fig 15 presents frequency-wavenumber spectrum analysis across 12 excitation conditions. The spectral visualization supports three findings. First, at the exact combined-resonance frequency (Ω/ω_0_ = 1.00, case 4), the spectral power is about 2.5 times higher than under off-resonance conditions, and the dominant wavenumber components concentrate near k≈±2.5 rad/m. This confirms that parametric excitation at (ω_1_ + ω_2_)/2 efficiently couples energy into the resonant modal pair. Second, as the excitation frequency moves away from resonance, the spectral power decreases with a Lorentzian-like profile; the half-width at half-maximum is approximately 0.08 ω_0_, which defines the practical tolerance of the resonance window. Third, as the tension-fluctuation amplitude increases, spectral peaks broaden and secondary harmonics appear. At γ ≈ 0.20, higher-order wavenumber components emerge, signaling the onset of strong nonlinear modal interaction. Thus, the frequency-wavenumber spectrum provides a direct diagnostic for localization intensity and pattern type.

The same spectral analysis also supports the discrimination between true traveling patterns and standing waves with slowly drifting nodes. A true traveling pattern produces an imbalance between positive and negative wavenumber branches and a consistent phase-velocity sign. In contrast, a drifting standing wave caused by weak boundary asymmetry produces nearly symmetric spectral peaks with slow low-frequency modulation. This diagnostic is therefore used together with phase unwrapping and energy-flux analysis to classify the pattern topology.

## 4. Discussion

The numerical investigations presented in Sections 3.1–3.12 provide evidence supporting the proposed framework for energy localization and spatiotemporal pattern evolution in parametrically excited thin-film structures. This discussion synthesizes the key findings, clarifies their physical interpretation, defines the validity limits of the model, and identifies implications for space-structure design and vibration control.

### 4.1 Unified mechanism of energy localization

The results demonstrate that energy localization in spatial thin-film structures under parametric excitation emerges from three coupled mechanisms: (1) parametric-resonance-induced modal instability, (2) nonlinear geometric stiffness coupling, and (3) scale-dependent self-organization. The combined resonance condition Ω=(ω_1_ + ω_2_)/2 provides the primary energy-injection pathway by transferring energy from the time-varying tension field into a specific modal pair. The tension threshold γ > 0.20 represents the point where the cycle-averaged parametric gain exceeds damping and weak nonlinear spreading. After this threshold is crossed, nonlinear saturation prevents unbounded growth and confines the response into a localized attractor. The nonlinear modal coupling strength κ then determines the pattern topology. The critical value κc ≈ 0.375 marks a phase-slip bifurcation: below it, the coupled modal phases remain locked and form stationary breathers; above it, phase locking is lost and symmetry-broken traveling patterns appear.

### 4.2 Scale invariance and predictive modeling

The proportional relationship between pattern spatial period and structural size (λ/L ≈ 0.28) is significant for scaled testing and modular design, but its domain of validity must be stated carefully. The relationship was obtained for square membranes with comparable in-plane dimensions and matched nondimensional parameters. Under these conditions, the intrinsic wavelength is selected by local dynamic equilibrium rather than by absolute size, allowing sub-scale tests to predict full-scale behavior. For highly anisotropic membranes, however, the selected wavelength is expected to depend on the vector wavenumber, anisotropic pretension, and boundary compliance. Therefore, scaled prototypes remain valid when geometric similarity and nondimensional excitation conditions are preserved, while extremely high-aspect-ratio sails require an anisotropic extension of the scaling law.

### 4.3 Pattern control and engineering applications

The pattern-selection mechanism provides a direct route from understanding localization to controlling it. Modal energy maps and energy-flux density fields identify where energy is injected, where it accumulates, and along which paths it travels. Passive dampers should be placed near velocity antinodes or recurrent hot spots to increase local dissipation without globally suppressing useful low-order modes. Local stiffeners should be arranged along predicted hot-spot boundaries or weak-stiffness regions to shift modal overlap integrals and reduce κ below the traveling-pattern threshold. Active actuators should be located at points with high modal controllability and observability so that small tension or curvature corrections can move the operating point away from γc and κc. In this way, the framework does not merely observe patterns; it supplies a quantitative placement rule for stiffeners, dampers, and active control elements.

### 4.4 Implications for space mission design

The findings also have implications for mission design. The tension threshold γ > 0.20 provides a practical constraint for on-orbit pretension management: normal thermal cycling and attitude maneuvers should be kept below this level with safety margins for detuning drift, support compliance, and manufacturing uncertainty. The stability results show that localization should not be treated as a rapidly decaying transient; once formed, it may persist as an attractor unless the operating point is moved out of the attraction basin. Frequency-dependent damping and material memory effects would modify this picture by introducing phase-lagged dissipation and history-dependent stiffness. Such effects generally shift resonance tongues, broaden or narrow the instability band, and can either suppress or enhance localization depending on their phase relative to the parametric input. Future mission-level models should therefore include viscoelastic constitutive terms, creep of the film material, and temperature-dependent natural frequencies.

### 4.5 Validity under frequency drift, frame flexibility, and material memory

The proposed theoretical model can accommodate gradual frequency drift by allowing the detuning parameter in the slow-flow equations to vary slowly with temperature and aging. If the drift rate is small compared with the envelope time scale, the response follows a quasi-static sequence of resonance tongues; localization strengthens when the drift moves the system toward the combined-resonance ridge and weakens when it moves away. Rapid thermal snaps or attitude maneuvers violate the slow-drift assumption and must be evaluated with the full finite element solver. Frame flexibility and material memory enter the same framework by modifying the modal frequencies, damping kernel, and coupling coefficients. In practical terms, these effects should be represented by a coupled membrane-frame model and, for viscoelastic films, by frequency-dependent damping or hereditary constitutive terms.

### 4.6 Practical implementation and future experimental validation

A realistic implementation path consists of three steps. First, a ground demonstrator should reproduce the modal generalized forcing of orbital excitation by using distributed thermal loading, edge piezoelectric actuators, or synchronized cable-tension modulation. Second, measured mode shapes, residual stresses, support compliance, and thickness maps should be imported into the finite element model to update the predicted γc and κc thresholds. Third, the resulting energy-flux and modal-participation maps should be used to place dampers, stiffeners, or active actuators before in-orbit operation. This workflow connects the theoretical localization mechanism to disturbance-tolerant design and provides a clear experimental-validation plan for future work.

## 5. Conclusion

This study establishes an integrated framework combining nonlinear thin-shell modeling, multiple-scales perturbation theory, Floquet-based stability interpretation, and high-fidelity finite element simulations to explain energy localization and spatiotemporal pattern evolution in parametrically excited spatial thin-film structures. The analysis identifies the combined resonance condition Ω=(ω_1_ + ω_2_)/2 and the tension fluctuation threshold γ > 0.20 as criteria for pronounced localization. It further shows that nonlinear modal coupling strength κ is the immediate control parameter for pattern topology, with κc ≈ 0.375 ± 0.025 separating stationary breathers from traveling patterns. The scale relationship λ/L ≈ 0.28 is verified for square and near-square membranes and provides a useful basis for scaled testing, while anisotropic geometries require additional aspect-ratio correction. Stability verification over 2000 excitation cycles indicates that both stationary and traveling localized patterns behave as persistent attractors within the simulated horizon, although mission-level extrapolation must account for frequency drift, material memory, support-frame aging, and thermal-stress relaxation. The revised framework also clarifies how localization analysis can be translated into engineering control: modal energy and energy-flux maps indicate where stiffeners, passive dampers, or active actuators should be placed to move the system away from damaging γ and κ regimes. Future work will focus on ground experimental validation using distributed thermal or piezoelectric excitation, coupled membrane-frame testing, and closed-loop pretension control so that the proposed guidelines can be implemented in deployable aerospace structures.
